# In-vivo iron mapping in patients with Parkinson’s disease using deep learning-based susceptibility source separation MRI

**DOI:** 10.1038/s41531-026-01384-x

**Published:** 2026-05-27

**Authors:** Hyeong-Geol Shin, Kelly A. Mills, Ted M. Dawson, Taechang Kim, Jongho Lee, Xu Li, Peter van Zijl, Jannik Prasuhn

**Affiliations:** 1 F.M. Kirby Research Center for Functional Brain Imaging, Kennedy Krieger Institute, Baltimore, MD USA; 2 Department of Neurology, Johns Hopkins University School of Medicine, Baltimore, MD USA; 3 Neuroregeneration and Stem Cell Programs, Institute for Cell Engineering, Johns Hopkins University School of Medicine, Baltimore, MD USA; 4 Department of Physiology, Pharmacology and Therapeutics, Johns Hopkins University School of Medicine, Baltimore, MD USA; 5 Solomon H. Snyder Department of Neuroscience, Johns Hopkins University School of Medicine, Baltimore, MD USA; 6 Department of Electrical and Computer Engineering, Seoul National University, Seoul, Republic of Korea; 7 Division of MR Research, Department of Radiology, Johns Hopkins University School of Medicine, Baltimore, MD USA; 8 Section of Movement Disorders, Department of Neurology, University Medical Center Schleswig-Holstein, Campus Lübeck, Lübeck, Germany; 9 Institute of Neurogenetics, University of Lübeck, Lübeck, Germany; 10 Center for Brain, Behavior and Metabolism, University of Lübeck, Lübeck, Germany

**Keywords:** Computational biology and bioinformatics, Diseases, Neurology, Neuroscience

## Abstract

Parkinson’s disease (PD) involves pathological iron accumulation, yet MRI metrics, such as R_2_* or magnetic susceptibility (χ), lack mechanistic specificity because they convolve paramagnetic and diamagnetic sources. We applied an AI-assisted χ-separation framework that combines deep learning (DL)-based preprocessing with biophysical modeling to assess paramagnetic iron with enhanced specificity. Twenty-five PD patients and twenty-six matched controls underwent 3 T multi-parametric MRI. DL-based χ-separation (χ-separation_DL_) separated the paramagnetic susceptibility component (χ_para_; indicative of iron) from χ, revealing alterations undetected by established susceptibility-based methods: χ_para_ increased in dorsal premotor cortex ( +6.3%, P = 0.032) and substantia nigra pars compacta ( +10.2%, P = 0.024), χ_para_ in premotor cortex correlated with disease duration (r = 0.41; P = 0.045). DL-based preprocessing was not inferior for the differentiation between PD patients vs. controls compared to established optimization-based χ-separation, indicating the potential for AI-enhanced χ-separation to be applied within the scope of susceptibility imaging in PD.

## Introduction

Parkinson’s disease (PD) is the second most common neurodegenerative disorder and is defined pathologically by the progressive degeneration of dopaminergic neurons in the substantia nigra (SN)^1^. Among the molecular and cellular changes implicated in PD, abnormal iron accumulation has emerged as a central hallmark with pathogenic relevance^2^. Iron is essential for neuronal metabolism, but its dysregulation promotes oxidative stress, mitochondrial dysfunction, and neuroinflammation, thereby contributing to the selective vulnerability of dopaminergic neurons in PD^3^.

Given its central role in PD pathophysiology, iron has become a key target as a potential neuroimaging biomarker^4^. MRI-based approaches such as effective transverse relaxation rate (R_2_*) mapping and quantitative susceptibility mapping (QSM) have been widely used to estimate changes in regional iron content in vivo^4^. These methods have demonstrated utility in distinguishing patients with PD (PwPD) from healthy controls (HCs), characterizing prodromal stages, monitoring disease progression, and linking iron-related changes to clinical symptom burden^5–7^. However, despite their sensitivity to magnetic susceptibility (χ), both R_2_* and QSM are fundamentally limited by their lack of chemical specificity to iron, because diamagnetic sources (e.g., myelin and protein) are present in key neuroanatomical regions involved in PD pathogenesis and also contribute to the derived signal^8^.

In PwPD, pathological changes, such as iron accumulation^9^, demyelination^10^, and synucleinopathy^11^, may occur simultaneously. QSM quantifies the total ( = voxel-averaged) magnetic susceptibility (χ_QSM_) in a brain tissue, making it impossible to determine whether increases in χ_QSM_ reflect elevated paramagnetic sources, declined diamagnetic sources (e.g., myelin, *α*-synuclein), or both^12^. R_2_* is also influenced by both paramagnetic and diamagnetic sources, obscuring the specific role of iron^5^. These ambiguities restrict the interpretability and diagnostic precision of susceptibility-based biomarkers in PD, even in deep gray matter where myelin contributions can be non-negligible (e.g., substantia nigra)^13^.

To address this limitation, susceptibility source separation approaches were proposed to disentangle the contributions of paramagnetic (χ_para_) and diamagnetic (χ_dia_) sources to the MRI signal^14^. In particular, a recent source separation method, χ-separation (or chi-separation), models two complementary MRI parameters, frequency shifts and reversible transverse relaxation (R_2_′ = R_2_* − R_2_), as functions of χ_para_ and χ_dia_^15^. This approach has been validated in phantoms and ex-vivo tissue, and Shin et al. demonstrated its utility for mapping paramagnetic iron and diamagnetic myelin in pathological conditions such as multiple sclerosis^16,17^. By accounting for the diamagnetic component, χ-separation can more specifically characterize iron-related alterations (through χ_para_). As with QSM, however, χ-separation requires solving an ill-posed dipole inversion to estimate susceptibility from frequency shifts, which can introduce streaking artifacts even under a regularization framework, potentially biasing χ_para_ estimates. These effects can be exacerbated by motion^18,19^, which is commonly observed in MRI of PwPD, and may diminish clinical sensitivity. Because χ-separation relies on multiple observations (i.e., frequency shift and R_2_’), motion sensitivity of the relaxation measurements can further degrade image quality^20^. Acquiring multiple frequency shift maps from different head orientations can improve the conditioning of the dipole inversion, but such protocols are constrained by long scan times and patient burden with repeated reorientation^21^.

Recent studies have applied source separation methods (e.g., DECOMPOSE QSM, QSM-ARCS, APART-QSM) in PD and related synucleinopathies, highlighting their potential to capture disease-specific iron pathology^22,23^. In the present study, we extended this work to investigate PD-related alterations in paramagnetic iron by applying an AI-assisted χ-separation approach. This approach integrates deep learning (DL)-based preprocessing with a biophysical χ-separation model (χ-separation_DL_), producing an artifact-resistant map from single-orientation data. We compared iron-sensitive signals from χ-separation_DL_ (i.e., χ_para,DL_) against those from established *Morphology enabled dipole inversion* (MEDI)-based χ-separation (i.e., χ_para,MEDI_), QSM, and R_2_* metrics in a well-characterized cohort of patients with PD and matched HCs. The aims of this study were twofold: (i) to evaluate whether deep learning-based χ-separation improves the detection and interpretability of iron-related susceptibility alterations in cortical and subcortical gray matter of PwPD, as compared with established quantitative MRI metrics (QSM, R2*) and optimization-based χ-separation, and (ii) to assess the clinical relevance of these measures through associations with motor severity and disease duration.

## Results

### Demographics and clinical data

We included 25 PwPD (13 females, 12 males; age: 68.3 ± 6.9 years) and 26 age- and sex-matched HCs (11 females, 15 males; age: 66.2 ± 7.9 years). The PwPD cohort was predominantly in a mid-stage disease phase, with an average disease duration of 8.1 ± 4.8 years. Motor symptom severity was moderate overall, as reflected by a mean Movement Disorders Society - Unified Parkinson’s Disease Rating Scale (MDS-UPDRS) subscore III (MDS-UPDRS-III, assessed in medication off state) of 31.1 ± 14.1 points, an MDS-UPDRS subscore IV score of 2.2 ± 2.3 points, and a median Hoehn and Yahr stage of 2 (range: 1-4). Non-motor symptom burden was also moderate, with mean MDS-UPDRS subscore I and II scores of 9.8 ± 5.5 and 9.2 ± 6.3 points, respectively. Cognitive performance did not differ significantly between groups (Montreal Cognitive Assessment, MoCA, scores: 27.9 ± 1.69 in PwPD vs. 27.3 ± 3.40 in HCs; p = 0.45). However, PwPD showed significantly higher depressive symptomatology, with a mean Beck’s Depression Inventory-II (BDI-II) score of 7.7 ± 5.3 compared to 2.5 ± 5.5 in HCs (p = 0.001). A detailed summary of demographic and clinical characteristics is presented in Table 1.

**Table 1:** Demographic and clinical characteristics^a^ of the study cohort

	PwPD (*n* = 25)	HCs (*n* = 26)	*P*-value^c^
Sex [f/m]	13 / 12	11 / 15	0.579
Age [years]	68.3 ± 6.9	66.2 ± 7.9	0.316
Disease duration [years]	8.1 ± 4.8	N/A	–
MDS-UPDRS-I	9.8 ± 5.5	N/A	–
MDS-UPDRS-II	9.2 ± 6.3	N/A	–
MDS-UPDRS-III	31.1 ± 14.1	N/A	–
MDS-UPDRS-IV	2.2 ± 2.3	N/A	–
Hoehn and Yahr stage^b^	2 (1; 4)	N/A	–
LEDD [mg/d]	948.0 ± 469.6	N/A	–
Laterality of symptoms [n = left/right]	14/11	N/A	–
MoCA	27.9 ± 1.7	27.3 ± 3.4	0.428
BDI-II	7.7 ± 5.3	2.5 ± 5.5	0.001

*BDI-II, Beck's Depression Inventory (version 2), f* female, *HCs* healthy controls, *LEDD* levodopa-equivalent daily dosage, *m* male, *MDS-UPDRS* Movement Disorders Society - Unified Parkinson’s Disease Rating Scale, *MoCA* Montreal Cognitive Assessment, *N/A* not applicable, *PwPD* patients with Parkinson’s disease.

^a^All metrics are reported as mean ± standard deviation (if not stated otherwise).

^b^Median (minimum; maximum).

^c^*p*-values were calculated using Fisher’s exact test for categorical variables (sex) and Welch’s t-tests for continuous variables.

### χ-separationDL reveals region-specific susceptibility alterations in premotor cortices of patients with Parkinson’s disease

**Fig. 1:**
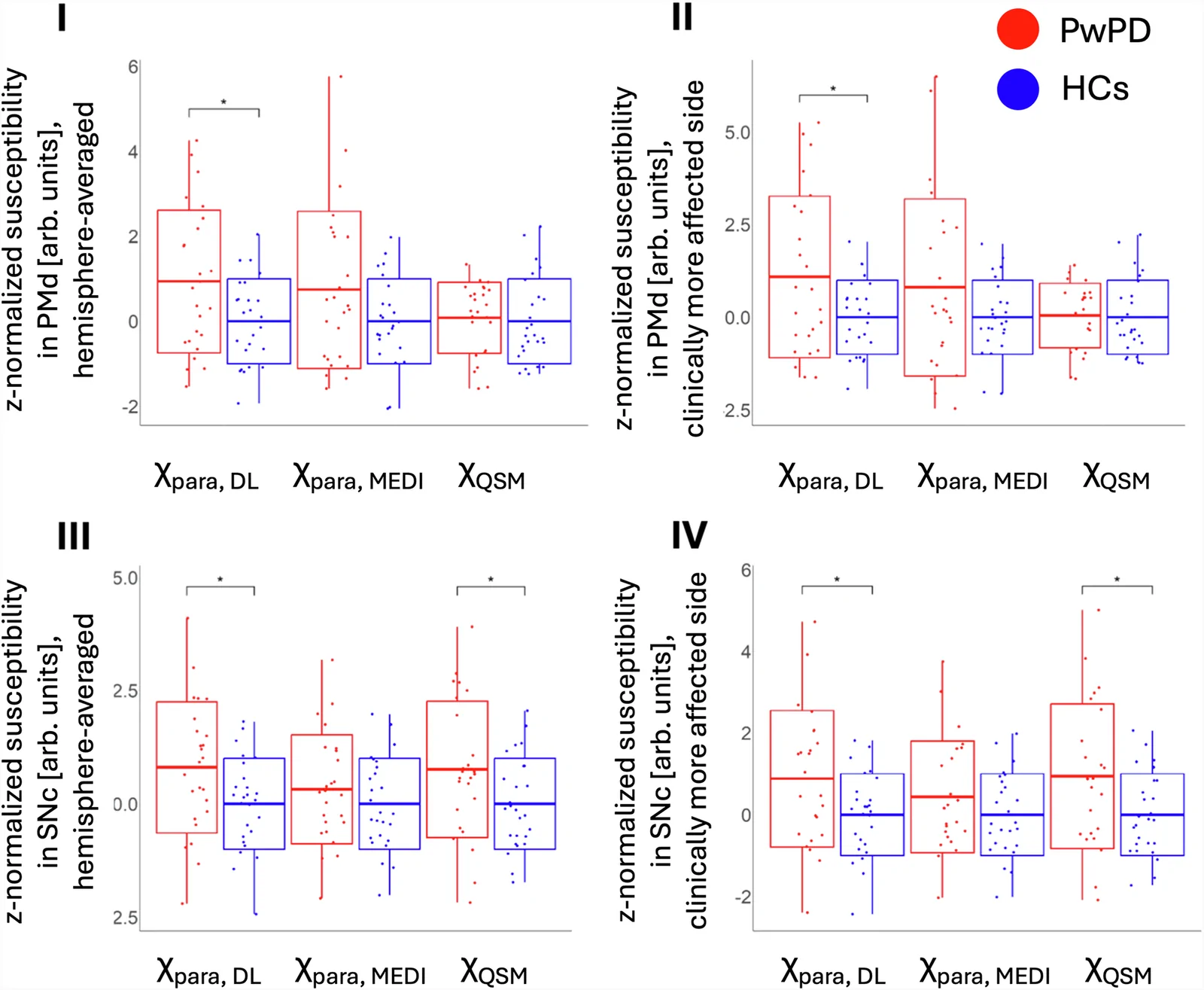
Group differences in z-normalized magnetic susceptibility across PMd and SNc in PwPD and HCs. Panels I–IV show box-and-whisker plots of z-normalized susceptibility values (arbitrary units) comparing PwPD (red) to HCs (blue). All values were z-normalized to the healthy control group for the respective region (HC mean = 0; SD = 1). Panels I–II display results for the PMd and Panels III–IV for the SNc. Panels I and III show hemisphere-averaged data, whereas Panels II and IV depict values for the clinically more affected side only. Each box represents the standard deviation after z-normalization, the horizontal line indicates the group mean, and whiskers denote the total range. Results are displayed for three susceptibility metrics: χ_para,DL_, χ_para,MEDI_, and χ_QSM_. Notably, χ_para,DL_ demonstrates greater sensitivity to group differences compared with the MEDI-based χ-separation and QSM approaches. arb. units: arbitrary units. PwPD: Patients with Parkinson’s disease. HC = healthy control. PMd: dorsal premotor cortex. SNc: substantia nigra pars compacta. QSM: quantitative susceptibility mapping, DL: deep learning-based reconstruction. MEDI: morphology-enabled dipole inversion. χ_para,DL_: paramagnetic component derived from deep-learning χ-separation; χ_para,MEDI_: paramagnetic component derived from MEDI-based χ-separation; χ_QSM:_ total magnetic susceptibility derived from QSM reconstruction.

Across the six *Human Motor Area Template* (HMAT)-defined motor regions (primary motor cortex, M1; primary somatosensory cortex, S1; supplementary motor area, SMA; pre-supplementary motor area, preSMA; dorsal premotor cortex, PMd; and ventral premotor cortex, PMv), hemisphere-averaged χ_QSM_ from QSM showed no significant group differences between PwPD and HCs. By contrast, hemisphere-averaged χ_para,DL_ was significantly increased in the PMd of PwPD ( +6.3%; 0.0254 ± 0.0026 ppm vs. 0.0239 ± 0.0016 ppm; F(1, 46) = 4.92; P = .032, false discovery-rate corrected P, P_FDR_ = .192). Bilaterally-averaged R_2_* also revealed significant increases in PwPD, with elevated values in the PMd ( +2.9%; 18.70 ± 1.03 s^-1^ vs. 18.18 ± 0.57 s^-1^; F(1, 44) = 5.93; P = .019, P_FDR_ = .114) and PMv ( +2.0%; 18.45 ± 0.74 s^-1^ vs. 18.08 ± 0.61 s^-1^; F(1, 44) = 4.48; P = .040, P_FDR_ = .120). A detailed summary of these findings is presented in Tables 2 and 3. The significant group comparisons are also presented as box plot diagrams in Fig. 1.

**Table 2:** Regional group comparisons of hemisphere-averaged cortical quantitative susceptibilities^a^ (in ppm)

		PwPD			HCs			F(df)^b^	*P*-value^b,c^ ∣P-value_FDR_
**χ**_**QSM**_	M1	0.003	±	0.008	0.000	±	0.012	F(1, 46) = 1.44	0.237 ∣ 0.711
	S1	−0.008	±	0.008	-0.009	±	0.012	F(1, 47) = 0.37	0.545 ∣ 0.817
	SMA	0.001	±	0.013	0.002	±	0.013	F(1, 47) = 0.07	0.794 ∣ 0.893
	preSMA	-0.003	±	0.015	-0.003	±	0.013	F(1, 46) = 0.01	0.940 ∣ 0.968
	PMd	-0.005	±	0.009	-0.005	±	0.011	F(1, 47) = 0.15	0.701 ∣ 0.878
	PMv	-0.003	±	0.007	-0.006	±	0.009	F(1, 46) = 2.17	0.148 ∣ 0.532
**χ**_**para,DL**_	M1	0.029	±	0.003	0.029	±	0.002	F(1, 44) = 0.12	0.732 ∣ 0.883
	S1	0.025	±	0.002	0.025	±	0.002	F(1, 46) = 0.18	0.670 ∣ 0.878
	SMA	0.030	±	0.003	0.029	±	0.003	F(1, 47) = 0.93	0.340 ∣ 0.765
	preSMA	0.027	±	0.003	0.027	±	0.003	F(1, 46) = 0.84	0.364 ∣ 0.765
	**PMd**	**0.025**	**±**	**0.003**	**0.024**	**±**	**0.002**	**F(1, 46)** **=** **4.92**	**0.032 ∣ 0.576**
	PMv	0.025	±	0.002	0.025	±	0.002	F(1, 46) = 0.20	0.660 ∣ 0.878
**χ**_**para,MEDI**_	M1	0.041	±	0.005	0.040	±	0.002	F(1, 40) = 0.25	0.617 ∣ 0.878
	S1	0.035	±	0.004	0.034	±	0.005	F(1, 46) = 0.22	0.639 ∣ 0.878
	SMA	0.040	±	0.006	0.040	±	0.008	F(1, 46) = 0.03	0.858 ∣ 0.913
	preSMA	0.037	±	0.006	0.037	±	0.009	F(1, 47) = 0.00	0.968 ∣ 0.968
	PMd	0.035	±	0.006	0.033	±	0.003	F(1, 46) = 3.14	0.083 ∣ 0.532
	PMv	0.034	±	0.004	0.033	±	0.003	F(1, 45) = 1.82	0.184 ∣ 0.552

*QSM* quantitative susceptibility mapping, *DL* deep learning-based reconstruction, *MEDI* morphology-enabled dipole inversion, *χ*_*QSM*_ total magnetic susceptibility derived from QSM, *χ*_*para,DL*_ paramagnetic component derived from deep-learning χ-separation, *χ*_*para,MEDI*_ paramagnetic component derived from MEDI-based χ-separation, *HCs* healthy controls, *PwPD* patients with Parkinson’s disease, *M1* primary motor cortex, *S1* primary somatosensory cortex, *SMA* supplementary motor area, *preSMA* pre-supplementary motor area, *PMd* dorsal premotor cortex, *PMv* ventral premotor cortex.

^a^Mean ± standard deviation of susceptibility parameters in predefined motor cortical regions of interest for patients with Parkinson’s disease (PwPD) and healthy controls (HCs).

^b^Reported statistics are F-values with corresponding degrees of freedom and *p*-values from univariate ANCOVAs (accounting for sex and age as covariates).

^c^Significant (uncorrected) *p*-values have been highlighted in bold.

**Table 3:** Cortical and subcortical R_2_* values^a^ (in s^-1^) in patients with Parkinson’s disease versus healthy controls: analyses of the clinically more affected hemisphere and hemisphere-averaged values

	cortical regions (hemisphere-averaged)								cortical regions (clinically more affected side)							
Region	PwPD			HCs			F(df)^b^	*P*-value ^b,c^ ∣ P-value_FDR_	PwPD				HCs		F(df)^b^	*P*-value ^b,c^ ∣ P-value_FDR_
M1	19.4	±	0.9	19.3	±	0.7	F(1, 44) = 0.25	0.622 ∣ 1.000	19.2	±	1.0	19.4	±	0.7	F(1, 43) = 0.23	0.636 ∣ 1.000
S1	18.0	±	0.9	17.9	±	0.5	F(1, 42) = 0.55	0.464 ∣ 1.000	17.9	±	1.2	17.9	±	0.5	F(1, 41) = 0.05	0.821 ∣ 1.000
SMA	18.8	±	0.9	18.6	±	0.8	F(1, 43) = 0.39	0.537 ∣ 1.000	18.8	±	1.0	18.6	±	0.8	F(1, 42) = 0.26	0.612 ∣ 1.000
preSMA	17.9	±	0.9	17.5	±	0.8	F(1, 44) = 1.89	0.177 ∣ 0.708	17.9	±	1.1	17.5	±	0.8	F(1, 43) = 1.67	0.203 ∣ 1.000
PMd	**18.7**	**±**	**1.0**	**18.2**	**±**	**0.6**	**F(1, 44)** **=** **5.93**	**0.019 ∣ 0.228**	18.4	±	1.1	18.2	±	0.6	F(1, 42) = 0.82	0.370 ∣ 1.000
PMv	**18.5**	**±**	**0.7**	**18.1**	**±**	**0.6**	**F(1, 44)** **=** **4.48**	**0.040 ∣ 0.240**	18.2	±	1.0	18.1	±	0.6	F(1, 42) = 0.14	0.712 ∣ 1.000
	**subcortical regions (hemisphere-averaged)**								**subcortical regions (clinically more affected side)**							
**Region**	**PwPD**			**HCs**			**F(df)** ^**b**^	**P-value** ^**b,c**^ **∣ P-value**_**FDR**_	**PwPD**			**HCs**			**F(df)** ^**b**^	**P-value** ^**b,c**^ **∣ P-value**_**FDR**_
Cau	23.4	±	2.4	23.2	±	2.2	F(1, 42) = 0.25	0.617 ∣ 1.000	23.4	±	2.4	23.2	±	2.2	F(1, 42) = 0.25	0.617 ∣ 1.000
Pu	29.2	±	5.0	29.1	±	4.2	F(1, 45) = 0.01	0.911 ∣ 1.000	29.2	±	5.0	29.1	±	4.2	F(1, 45) = 0.01	0.911 ∣ 1.000
GPe	36.3	±	4.3	36.8	±	3.3	F(1, 42) = 0.06	0.805 ∣ 1.000	36.3	±	4.3	36.8	±	3.3	F(1, 42) = 0.06	0.805 ∣ 1.000
NAcc	22.9	±	3.4	24.5	±	4.2	F(1, 44) = 1.86	0.179 ∣ 1.000	22.9	±	3.4	24.5	±	4.3	F(1, 44) = 1.86	0.179 ∣ 1.000
SNc	34.1	±	8.0	33.4	±	7.5	F(1, 46) = 0.28	0.597 ∣ 1.000	34.1	±	8.0	33.4	±	7.5	F(1, 46) = 0.28	0.597 ∣ 1.000
SNr	36.7	±	6.7	38.3	±	8.3	F(1, 46) = 0.19	0.665 ∣ 1.000	36.7	±	6.7	38.3	±	8.3	F(1, 46) = 0.19	0.665 ∣ 1.000
RN	31.8	±	5.8	32.9	±	5.8	F(1, 44) = 0.08	0.781 ∣ 1.000	31.8	±	5.8	32.9	±	5.8	F(1, 44) = 0.08	0.781 ∣ 1.000
VP	41.3	±	10.9	40.3	±	8.3	F(1, 45) = 0.18	0.675 ∣ 1.000	41.3	±	10.9	40.3	±	8.6	F(1, 45) = 0.18	0.675 ∣ 1.000
GPi	34.1	±	6.0	34.3	±	3.7	F(1, 43) = 0.02	0.878 ∣ 1.000	34.1	±	6.0	34.3	±	3.7	F(1, 43) = 0.02	0.878 ∣ 1.000
STN	34.9	±	6.6	35.4	±	4.3	F(1, 45) = 0.10	0.755 ∣ 1.000	34.9	±	6.6	35.4	±	4.3	F(1, 45) = 0.10	0.755 ∣ 1.000
Thal	19.5	±	1.3	20.1	±	2.7	F(1, 45) = 0.38	0.542 ∣ 1.000	19.5	±	1.3	20.1	±	2.7	F(1, 45) = 0.38	0.542 ∣ 1.000
Pulv	20.7	±	1.6	21.5	±	1.8	F(1, 42) = 2.32	0.135 ∣ 1.000	20.7	±	1.6	21.5	±	1.8	F(1, 42) = 2.32	0.135 ∣ 1.000

*HCs* healthy controls, *PwPD* patients with Parkinson’s disease, *R*_2_* effective transverse relaxation rate, *M1* primary motor cortex, *S1* primary somatosensory cortex, *SMA* supplementary motor area, *preSMA* pre-supplementary motor area, *PMd* dorsal premotor cortex, *PMv* ventral premotor cortex, *Cau* caudate nucleus, Pu putamen, *GPe* external globus pallidus, *GPi* internal globus pallidus, *NAcc* nucleus accumbens, *SNc* substantia nigra pars compacta, *SNr* substantia nigra pars reticulata, *RN* red nucleus, *VP* ventral pallidum, *STN* subthalamic nucleus, *Thal* thalamus, *Pulv* pulvinar.

^a^Values are reported as mean ± standard deviation in two types of regions: (i) hemisphere-averaged - the within-subject average across left and right hemispheres, and (ii) clinically more affected side - the hemisphere contralateral to the clinically more affected body side in PwPD (hemisphere-averaged hemispheres in HCs).

^b^Reported statistics are F-values with degrees of freedom (F(df)) from univariate ANCOVAs (accounting for sex and age as covariates), with two-sided p-values.

^c^Significant (uncorrected) p-values are highlighted in bold.

In the clinically more affected hemisphere, QSM again showed no significant findings. In contrast, χ_para,DL_ values were significantly elevated in the PMd on the clinically more affected hemisphere ( +7.1%; 0.0256 ± 0.0034 ppm vs. 0.0239 ± 0.0016 ppm; F(1, 45) = 4.45; P = .041, P_FDR_ = .246). R_2_* on the clinically more affected side revealed no significant changes in cortical regions of the clinically more affected hemisphere. A detailed summary of these findings is presented in Tables 3 and 4. The significant group comparisons are also presented as box plot diagrams in Fig. 1. The corresponding χ_dia_ results have been highlighted in Supplementary Table 1.

**Table 4:** Regional group comparisons of cortical quantitative susceptibilities^a^ (in ppm) on the clinically more affected hemisphere

		PwPD			HCs			F(df)^b^	P-value ^b,c^ ∣ P-value_FDR_
**χ**_**QSM**_	M1	0.003	±	0.009	0.000	±	0.012	F(1, 46) = 1.08	0.304 ∣ .912
	S1	-0.008	±	0.008	-0.009	±	0.012	F(1, 46) = 0.44	0.512 ∣ .864
	SMA	0.002	±	0.014	0.002	±	0.013	F(1, 46) = 0.04	0.844 ∣ .864
	preSMA	-0.003	±	0.019	-0.003	±	0.013	F(1, 45) = 0.03	0.864 ∣ .864
	PMd	-0.005	±	0.009	-0.005	±	0.011	F(1, 46) = 0.09	0.766 ∣ .864
	PMv	-0.004	±	0.007	-0.006	±	0.009	F(1, 46) = 1.70	0.198 ∣ .912
**χ**_**para,DL**_	M1	0.029	±	0.003	0.029	±	0.002	F(1, 42) = 0.05	0.830 ∣ .830
	S1	0.025	±	0.003	0.025	±	0.002	F(1, 46) = 0.79	0.380 ∣ .570
	SMA	0.030	±	0.003	0.029	±	0.003	F(1, 45) = 0.53	0.472 ∣ .570
	preSMA	0.027	±	0.003	0.027	±	0.003	F(1, 45) = 0.83	0.367 ∣ .570
	**PMd**	**0.026**	**±**	**0.003**	**0.024**	**±**	**0.002**	**F(1, 45)** **=** **4.45**	**0.041 ∣ .246**
	PMv	0.025	±	0.002	0.025	±	0.002	F(1, 46) = 0.10	0.756 ∣ .830
**χ**_**para,MEDI**_	M1	0.040	±	0.005	0.040	±	0.002	F(1, 38) = 0.02	0.878 ∣ .989
	S1	0.034	±	0.005	0.034	±	0.005	F(1, 44) = 0.00	0.989 ∣ .989
	SMA	0.040	±	0.007	0.040	±	0.008	F(1, 45) = 0.17	0.680 ∣ .989
	preSMA	0.037	±	0.007	0.037	±	0.009	F(1, 46) = 0.02	0.895 ∣ .989
	PMd	0.035	±	0.008	0.033	±	0.003	F(1, 45) = 2.48	0.123 ∣ .522
	PMv	0.035	±	0.006	0.033	±	0.003	F(1, 45) = 1.91	0.174 ∣ .522

*QSM* quantitative susceptibility mapping, *DL* deep learning-based reconstruction, *MEDI* morphology-enabled dipole inversion, *χ*_*QSM*_ total magnetic susceptibility derived from QSM, *χ*_*para,DL*_ paramagnetic component derived from deep-learning χ-separation, *χ*_*para,MEDI*_ paramagnetic component derived from MEDI-based χ-separation, *HCs* healthy controls, *PwPD* patients with Parkinson’s disease, *M1* primary motor cortex, *S1* primary somatosensory cortex, *SMA* supplementary motor area, *preSMA* pre-supplementary motor area, *PMd* dorsal premotor cortex, *PMv* ventral premotor cortex.

^**a**^Mean ± standard deviation of QSM-derived parameters in predefined motor cortical regions of interest for patients with Parkinson’s disease (PwPD) and healthy controls (HCs).

^b^Reported statistics are F-values with corresponding degrees of freedom p-values from univariate ANCOVAs (accounting for sex and age as covariates).

^c^Significant (uncorrected) p-values have been highlighted in bold.

### χ-separation DL reveals complementary susceptibility changes in deep gray matter of patients with Parkinson’s disease

Across the twelve *Multi-contrast susceptibility-based subcortical atlas* (MuSus-100) deep gray matter regions (caudate nucleus, Cau; putamen, Pu; external globus pallidus, GPe; nucleus accumbens, NAcc; substantia nigra pars compacta, SNc; substantia nigra pars reticulata, SNr; red nucleus, RN; ventral pallidum, VP; internal globus pallidus, GPi; subthalamic nucleus, STN; thalamus, Thal; and pulvinar, Pulv), χ_para,DL_ was significantly elevated in the SNc of PwPD ( +10.2%; 0.1322 ± 0.0220 ppm vs. 0.1200 ± 0.0152 ppm; F(1, 47) = 5.42; P = .024, P_FDR_ = .288). In the same area, χ_QSM_ showed higher values in PwPD than HCs ( +16.5%; PwPD: 0.1409 ± 0.0395 ppm vs. HCs: 0.1210 ± 0.0262 ppm; F(1, 47) = 4.08; P = .049). No significant group effects were detected by R_2_* mapping in any deep gray matter ROI, when bilaterally averaged. A detailed summary of these findings is presented in Tables 3 and 5. The significant group comparisons are also presented as box plot diagrams in Fig. 1. On the clinically more affected hemisphere, χ_para,DL_ was significantly elevated in the SNc on the clinically more affected side ( +11.2%; PwPD: 0.1334 ± 0.0254 ppm vs. 0.1200 ± 0.0152 ppm; F(1, 46) = 5.16; P = .028, P_FDR_ = .336). A significant χ_QSM_ difference was observed in the same region, where PwPD showed higher values than HCs ( +20.5%; PwPD: 0.1458 ± 0.0465 ppm vs. HCs: 0.1210 ± 0.0262 ppm; F(1, 46) = 5.21; P = .027). No significant differences were observed for R_2_* mapping. A detailed summary of these findings is presented in Tables 3 and 6. The significant group comparisons are also presented as box plot diagrams in Fig. 1. The corresponding χ_dia_ results have been highlighted in Supplementary Table 2.

**Table 5:** Regional group comparisons of hemisphere-averaged subcortical quantitative susceptibility metrics^a^ (in ppm)

		PwPD			HCs			F(df)^b^	P-value ^b,c^ ∣ P-value_FDR_
**χ**_**QSM**_	Cau	0.061	±	0.023	0.059	±	0.021	F(1, 47) = 0.00	0.991 ∣ 0.991
	Pu	0.050	±	0.023	0.061	±	0.030	F(1, 46) = 2.38	0.130 ∣ 0.435
	GPe	0.127	±	0.027	0.118	±	0.017	F(1, 44) = 2.20	0.145 ∣ 0.435
	NAcc	0.027	±	0.020	0.020	±	0.023	F(1, 46) = 0.53	0.469 ∣ 0.563
	**SNc**	**0.141**	**±**	**0.040**	**0.121**	**±**	**0.026**	**F(1, 47)** **=** **4.08**	**0.049 ∣ 0.401**
	SNr	0.132	±	0.021	0.141	±	0.031	F(1, 43) = 0.89	0.351 ∣ 0.563
	RN	0.102	±	0.018	0.092	±	0.030	F(1, 42) = 1.38	0.247 ∣ 0.494
	VP	0.117	±	0.053	0.118	±	0.033	F(1, 43) = 0.02	0.896 ∣ 0.977
	GPi	0.084	±	0.025	0.080	±	0.020	F(1, 46) = 0.58	0.451 ∣ 0.563
	STN	0.090	±	0.026	0.103	±	0.025	F(1, 42) = 3.54	0.067 ∣ 0.402
	Thal	-0.010	±	0.011	-0.015	±	0.015	F(1, 47) = 1.68	0.202 ∣ 0.435
	Pulv	0.019	±	0.014	0.017	±	0.021	F(1, 47) = 0.55	0.462 ∣ 0.563
**χ**_**para,DL**_	Cau	0.065	±	0.011	0.064	±	0.006	F(1, 43) = 0.01	0.927 ∣ 0.980
	Pu	0.079	±	0.018	0.083	±	0.013	F(1, 45) = 1.26	0.267 ∣ 0.720
	GPe	0.132	±	0.015	0.128	±	0.006	F(1, 44) = 1.05	0.312 ∣ 0.720
	NAcc	0.051	±	0.012	0.049	±	0.010	F(1, 47) = 0.01	0.939 ∣ 0.980
	**SNc**	**0.132**	**±**	**0.022**	**0.120**	**±**	**0.015**	**F(1, 47)** **=** **5.42**	**0.024 ∣ 0.288**
	SNr	0.138	±	0.014	0.137	±	0.017	F(1, 45) = 0.38	0.541 ∣ 0.812
	RN	0.101	±	0.020	0.103	±	0.016	F(1, 44) = 0.23	0.631 ∣ 0.860
	VP	0.131	±	0.040	0.130	±	0.015	F(1, 42) = 0.00	0.980 ∣ 0.980
	GPi	0.112	±	0.010	0.110	±	0.009	F(1, 43) = 0.68	0.415 ∣ 0.720
	STN	0.110	±	0.018	0.115	±	0.018	F(1, 45) = 0.76	0.388 ∣ 0.720
	Thal	0.026	±	0.003	0.026	±	0.003	F(1, 46) = 0.41	0.527 ∣ 0.812
	Pulv	0.043	±	0.006	0.045	±	0.009	F(1, 47) = 0.74	0.392 ∣ 0.720
**χ**_**para,MEDI**_	Cau	0.075	±	0.012	0.078	±	0.012	F(1, 43) = 0.57	0.453 ∣ 1.000
	Pu	0.096	±	0.028	0.098	±	0.024	F(1, 46) = 0.19	0.661 ∣ 1.000
	GPe	0.151	±	0.027	0.151	±	0.016	F(1, 43) = 0.04	0.850 ∣ 1.000
	NAcc	0.062	±	0.018	0.063	±	0.018	F(1, 46) = 0.25	0.620 ∣ 1.000
	SNc	0.154	±	0.044	0.142	±	0.036	F(1, 47) = 1.24	0.271 ∣ 1.000
	SNr	0.158	±	0.035	0.169	±	0.041	F(1, 46) = 0.56	0.458 ∣ 1.000
	RN	0.119	±	0.030	0.124	±	0.024	F(1, 44) = 0.22	0.638 ∣ 1.000
	VP	0.167	±	0.060	0.153	±	0.036	F(1, 44) = 0.66	0.421 ∣ 1.000
	GPi	0.129	±	0.028	0.130	±	0.026	F(1, 46) = 0.00	0.995 ∣ 1.000
	STN	0.129	±	0.035	0.138	±	0.025	F(1, 45) = 1.13	0.294 ∣ 1.000
	Thal	0.035	±	0.006	0.035	±	0.009	F(1, 46) = 0.00	0.945 ∣ 1.000
	Pulv	0.058	±	0.010	0.059	±	0.012	F(1, 47) = 0.06	0.810 ∣ 1.000

^**a**^All metrics are reported as mean ± standard deviation.

^b^p-values were obtained using univariate ANCOVA (accounting for sex and age as covariates) per region and metric.

^c^Significant (uncorrected) p-values have been highlighted in bold.

*QSM* quantitative susceptibility mapping, *DL* deep learning-based reconstruction, *MEDI* morphology-enabled dipole inversion, *χ*_*QSM*_ total magnetic susceptibility derived from QSM, *χ*_*para,DL*_ paramagnetic component derived from deep-learning χ-separation, *χ*_*para,MEDI*_ paramagnetic component derived from MEDI-based χ-separation, *HCs* healthy controls, *PwPD* patients with Parkinson’s disease, *Cau* caudate nucleus, *Pu* putamen, *GPe* external globus pallidus, *GPi* internal globus pallidus, *NAcc* nucleus accumbens, *SNc* substantia nigra pars compacta, *SNr* substantia nigra pars reticulata, *RN* red nucleus, *VP* ventral pallidum, *STN* subthalamic nucleus, *Thal* thalamus, *Pulv* pulvinar.

**Table 6:** Regional group comparisons of subcortical quantitative susceptibility metrics^a^ (in ppm) on the clinically more affected hemisphere

		PwPD			HCs			F(df)^b^	P-value ^b,c^ ∣ P-value_FDR_
**χ**_**QSM**_	Cau	0.061	±	0.025	0.059	±	0.021	F(1, 46) = 0.01	0.918 ∣ 0.945
	Pu	0.053	±	0.028	0.061	±	0.030	F(1, 46) = 1.16	0.287 ∣ 0.430
	GPe	0.128	±	0.029	0.118	±	0.017	F(1, 43) = 2.71	0.107 ∣ 0.430
	NAcc	0.023	±	0.020	0.020	±	0.023	F(1, 45) = 0.00	0.945 ∣ 0.945
	**SNc**	**0.146**	**±**	**0.047**	**0.121**	**±**	**0.026**	**F(1, 46)** **=** **5.21**	**0.027 ∣ 0.324**
	SNr	0.138	±	0.036	0.141	±	0.031	F(1, 45) = 0.08	0.781 ∣ 0.936
	RN	0.102	±	0.025	0.092	±	0.030	F(1, 42) = 1.46	0.234 ∣ 0.430
	VP	0.124	±	0.049	0.118	±	0.033	F(1, 41) = 0.19	0.668 ∣ 0.891
	GPi	0.083	±	0.028	0.080	±	0.020	F(1, 45) = 0.47	0.496 ∣ 0.744
	STN	0.090	±	0.030	0.103	±	0.025	F(1, 42) = 2.42	0.127 ∣ 0.430
	Thal	-0.010	±	0.011	-0.015	±	0.015	F(1, 46) = 1.75	0.193 ∣ 0.430
	Pulv	0.019	±	0.014	0.017	±	0.021	F(1, 46) = 0.51	0.478 ∣ 0.744
**χ**_**para,DL**_	Cau	0.065	±	0.012	0.064	±	0.006	F(1, 42) = 0.01	0.909 ∣ 0.983
	Pu	0.079	±	0.019	0.083	±	0.013	F(1, 44) = 1.48	0.230 ∣ 0.460
	GPe	0.133	±	0.015	0.128	±	0.006	F(1, 43) = 1.58	0.215 ∣ 0.460
	NAcc	0.049	±	0.013	0.049	±	0.010	F(1, 46) = 0.23	0.631 ∣ 0.757
	**SNc**	**0.133**	**±**	**0.025**	**0.120**	**±**	**0.015**	**F(1, 46)** **=** **5.16**	**0.028 ∣ 0.336**
	SNr	0.141	±	0.017	0.137	±	0.017	F(1, 45) = 1.26	0.268 ∣ 0.460
	RN	0.098	±	0.017	0.103	±	0.016	F(1, 42) = 0.83	0.368 ∣ 0.552
	VP	0.130	±	0.044	0.130	±	0.015	F(1, 41) = 0.00	0.983 ∣ 0.983
	GPi	0.114	±	0.015	0.110	±	0.009	F(1, 43) = 1.73	0.195 ∣ 0.460
	STN	0.113	±	0.023	0.115	±	0.018	F(1, 45) = 0.11	0.743 ∣ 0.811
	Thal	0.026	±	0.003	0.026	±	0.003	F(1, 45) = 0.17	0.680 ∣ 0.757
	Pulv	0.042	±	0.008	0.045	±	0.009	F(1, 46) = 1.74	0.194 ∣ 0.460
**χ**_**para,MEDI**_	Cau	0.078	±	0.016	0.078	±	0.012	F(1, 43) = 0.00	0.992 ∣ 0.992
	Pu	0.093	±	0.026	0.098	±	0.024	F(1, 44) = 0.93	0.340 ∣ 0.816
	GPe	0.151	±	0.026	0.151	±	0.016	F(1, 42) = 0.00	0.987 ∣ 0.992
	NAcc	0.055	±	0.015	0.063	±	0.018	F(1, 44) = 3.59	0.065 ∣ 0.780
	SNc	0.158	±	0.049	0.142	±	0.036	F(1, 46) = 2.00	0.164 ∣ 0.816
	SNr	0.166	±	0.041	0.169	±	0.041	F(1, 46) = 0.00	0.956 ∣ 0.992
	RN	0.120	±	0.033	0.124	±	0.024	F(1, 43) = 0.12	0.729 ∣ 0.992
	VP	0.166	±	0.063	0.153	±	0.036	F(1, 43) = 0.68	0.416 ∣ 0.816
	GPi	0.129	±	0.031	0.130	±	0.026	F(1, 45) = 0.00	0.975 ∣ 0.992
	STN	0.133	±	0.041	0.138	±	0.025	F(1, 45) = 0.28	0.597 ∣ 0.992
	Thal	0.033	±	0.005	0.035	±	0.009	F(1, 45) = 0.46	0.499 ∣ 0.856
	Pulv	0.056	±	0.013	0.059	±	0.012	F(1, 46) = 0.30	0.588 ∣ 0.992

^a^All metrics are reported as mean ± standard deviation.

^b^p-values were obtained using univariate ANCOVA (accounting for sex and age as covariates) per region and metric. QSM: quantitative susceptibility mapping.

^c^Significant (uncorrected) p-values have been highlighted in bold.

*χ*_*para*_ paramagnetic susceptibility component, *DL* deep learning-based reconstruction, *MEDI* morphology-enabled dipole inversion, *HCs* healthy controls, *PwPD* patients with Parkinson’s disease, *Cau* caudate nucleus, *Pu* putamen, *GPe* external globus pallidus, *GPi* internal globus pallidus, *NAcc* nucleus accumbens, *SNc* substantia nigra pars compacta, *SNr* substantia nigra pars reticulata, *RN* red nucleus, *VP* ventral pallidum, *STN* subthalamic nucleus, *Thal* thalamus, *Pulv* pulvinar.

### Regional χ-separationDL measures correlate with disease duration and motor symptoms

For the hemisphere-averaged analyses, no statistically significant correlations were observed for χ_QSM_ or R_2_* values in the HMAT-defined cortical motor regions. χ_para,DL_ values in the PMd did not correlate significantly with any clinical measure when averaged across hemispheres. No further significant relationships were identified in other hemisphere-averaged subcortical ROIs.

**Fig. 2:**
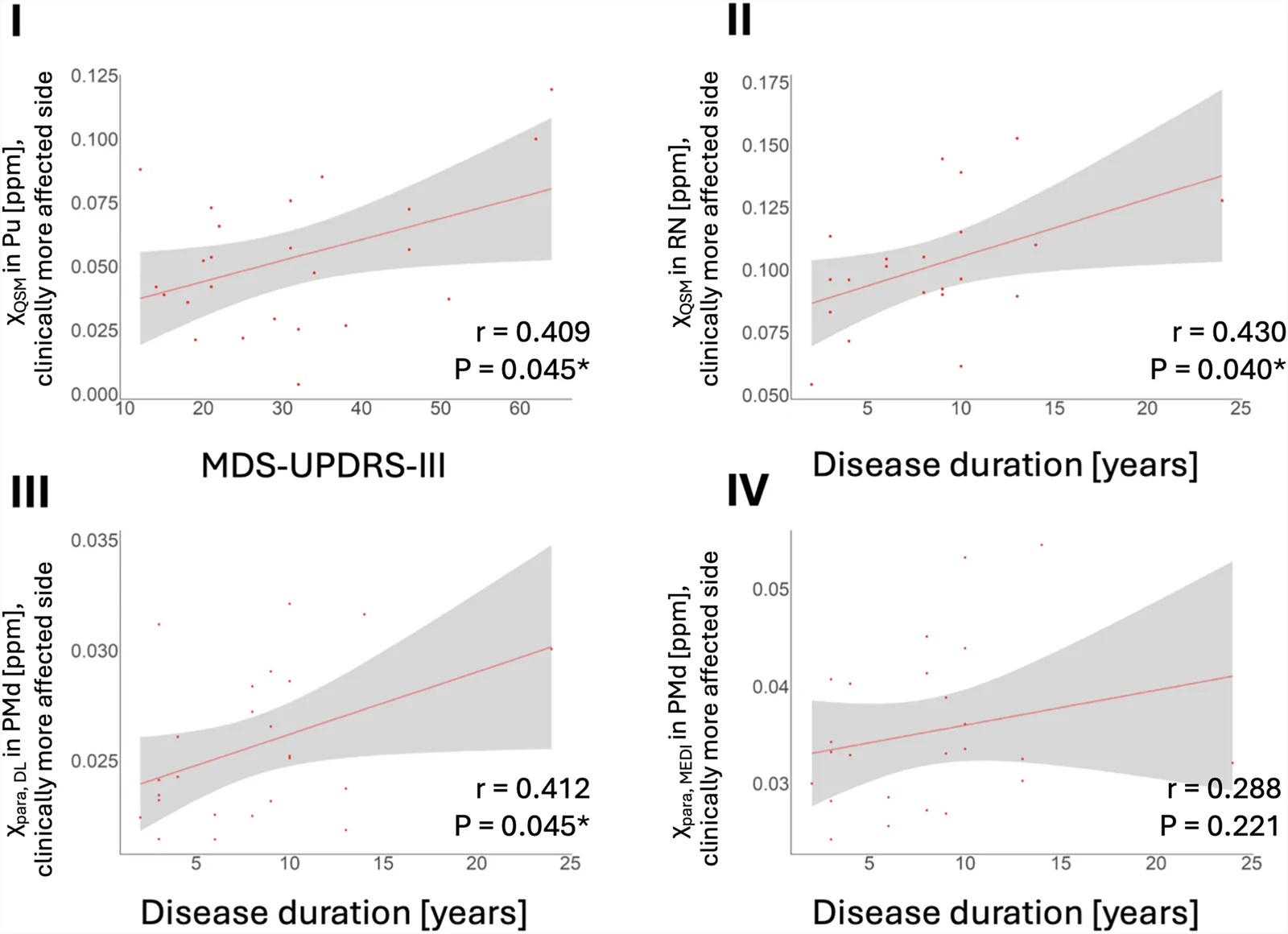
Correlation analyses between susceptibility metrics (χ_QSM_, and χ_para_ from χ-separation_DL_ and χ-separation_MEDI_) and clinical parameters in patients with Parkinson’s disease. In Panel I, χ_QSM_ in the Pu has been shown as a function of MDS-UPDRS-III. In panel II, χ_QSM_ in the RN has been shown as a function of the disease duration. Panel III depicts the significant correlation of the χ_para,DL_ in the PMd as a function of the disease duration. In contrast, Panel IV shows the non-significant χ_para,MEDI_ in the PMd susceptibility values as a function of the disease duration. Gray shading represents 95% confidence intervals of the fitted regression lines. * indicate significant findings. MDS-UPDRS-III: Movement Disorder Society-sponsored revision of the Unified Parkinson’s Disease Rating Scale, part III (motor examination). PD: Parkinson’s disease. PMd: dorsal premotor cortex. ppm: parts per million. Pu: putamen. RN: red nucleus. DL: deep learning-based reconstruction. MEDI: morphology-enabled dipole inversion. χ_para,DL_: paramagnetic component derived from deep-learning χ-separation. χ_para,MEDI_: paramagnetic component derived from MEDI-based χ-separation. χ_QSM:_ total magnetic susceptibility derived from QSM reconstruction.

In the clinically more affected hemisphere, χ-separation_DL_ metrics in the cortical regions demonstrated several associations with clinical measures: In the PMd, χ_para,DL_ values correlated significantly with disease duration (r = 0.412; 95% CI, 0.010 to 0.699; P = .045), while showing a trend-level association with MDS-UPDRS-III scores (r = 0.358; 95% CI, -0.053 to 0.665; P = .086). In the subcortical regions on the clinically more affected hemisphere, QSM values in the Pu correlated significantly with MDS-UPDRS-III (r = 0.409; 95% CI, 0.007 to 0.697; P = .047), while QSM in the RN was significantly associated with disease duration (r = 0.430; 95% CI, 0.022 to 0.716; P = .040) and showed a trend-level correlation with MDS-UPDRS-III (r = 0.360; P = .091). No significant correlations were observed for χ_para,DL_ in the SNc, or for R_2_* values in any subcortical region of the clinically more affected hemisphere. The graphical representation of the correlation analyses is shown in Fig. 2.

## Discussion

This study applied an AI-assisted χ-separation approach to assess paramagnetic iron in PwPD. Conventional iron-sensitive methods, such as χ_QSM_ and R_2_*, are widely used but remain biologically non-specific because paramagnetic and diamagnetic sources are convolved^24,25^.

By integrating deep learning-based preprocessing with a biophysical model of magnetic susceptibility and reversible transverse relaxation (R_2_′), χ-separation yields paramagnetic (χ_para_) maps that offer biologically interpretable insights into PD-related iron alterations with improved robustness to confounding artifacts^24,25^.

Our findings demonstrate that χ-separation_DL_ reveals disease-relevant elevations in paramagnetic susceptibility, indicative of iron accumulation, in both cortical and subcortical regions that conventional QSM or relaxation-based metrics did not capture. In the SNc, both χ_para,DL_ and χ_QSM_ increased in PwPD, consistent with pathological iron deposition and in agreement with prior source separation work^26^. In the PMd, χ_para,DL_ was significantly elevated (in bilateral averages and on the clinically more affected hemisphere) and correlated with disease duration (on the clinically more affected side), suggesting that motor-cortical iron accumulation may serve as a marker of disease progression and aligning with reports of increased motor-cortical iron, e.g., in motor neuron disease^27^. These effects were not evident in χ_QSM_, underscoring the value of source-specific mapping in disentangling overlapping pathological processes.

**Fig. 3:**
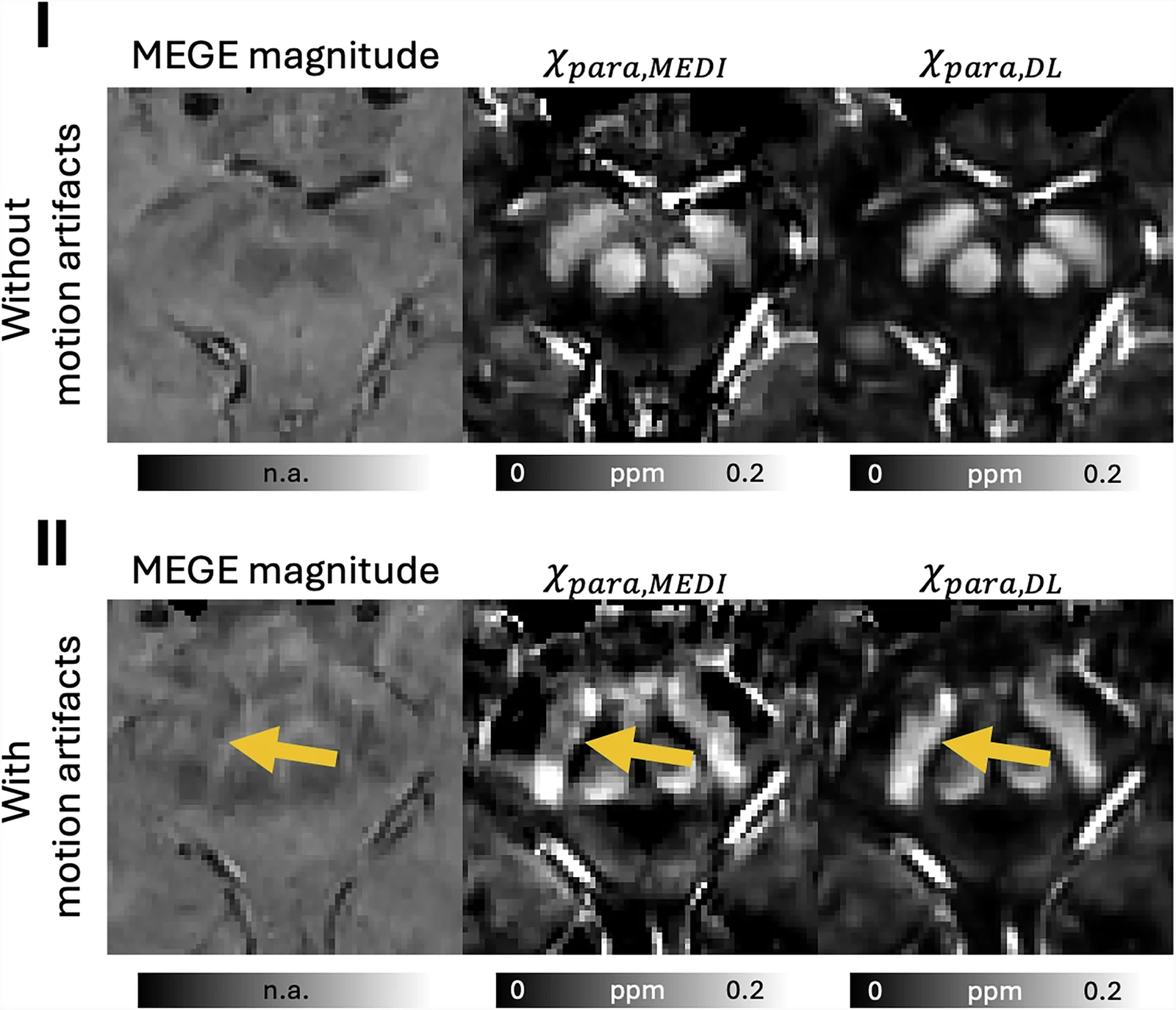
Paramagnetic susceptibility maps from χ-separation_MEDI_ and χ-separation_DL_ (χ_para,MEDI_ and χ_para,DL_) in patients with Parkinson’s disease, without (Panel I) and with (Panel II) visible head motion artifacts in the MEGE magnitude images. In the no-motion case (Panel I), χ_para,MEDI_ and χ_para,DL_ show comparable contrast. In the motion-affected scan (Panel II), the χ_para,MEDI_ map shows artifactual hypointensities in the middle of substantia nigra (orange arrows), whereas the χ_para,DL_ map displays preserved boundaries of substantia nigra, demonstrating improved motion robustness. χ-separation_MEDI_: MEDI-based χ-separation algorithm. χ-separation_DL_: deep learning-based χ-separation algorithm. MEGE: multi-echo gradient echo. χ_para,MEDI_: paramagnetic component derived from MEDI-based χ-separation. χ_para,DL_: paramagnetic component derived from deep-learning χ-separation.

Direct comparison between χ-separation_DL_ and χ-separation_MEDI_ further supports that DL-based preprocessing is not inferior to established preprocessing methods. χ-separation_DL_ consistently revealed cortical (PMd) and subcortical (SNc) group differences that χ-separation_MEDI_ did not capture. These findings suggest that DL-enabled preprocessing provides a viable alternative to ensure the biological interpretability and sensitivity of χ-separation, particularly in regions where subtle disease-related changes may be masked by streaking or motion artifacts in conventional pipelines (Fig. 3). The dissociation between χ-separation findings and conventional QSM or R_2_* underscores the limitations of total susceptibility and relaxation metrics and highlights the potential value of biophysically informed, AI-assisted reconstruction methods in biologically complex neurodegenerative conditions.

A discrepancy was observed in SNc, where χ_QSM_ and χ_para,DL_ revealed significant group differences between PwPD and HCs, whereas χ_para,MEDI_ did not. This finding is technically and biologically plausible. QSM reflects the net magnetic susceptibility within a voxel and therefore integrates contributions from both paramagnetic (e.g., iron) and diamagnetic components (e.g., myelin). As a result, disease-related combined iron accumulation and/or demyelination can therefore contribute to the elevated χ_QSM_ in PwPD vs. HCs. In contrast, χ-separation explicitly disentangles paramagnetic and diamagnetic contributions by jointly modeling frequency shifts and reversible transverse relaxation (R2′). In small, iron-rich nuclei such as the SNc pars compacta, this inversion problem is particularly sensitive to noise, partial volume effects, and motion-related artifacts, which can reduce statistical sensitivity of conventional, optimization-based χ-separation. Under these conditions, χ-separation may underestimate or fail to detect paramagnetic differences that remain visible in total susceptibility maps. This observation highlights that χ_QSM_ and χ-separation provide complementary information: χ_QSM_ may offer higher sensitivity to combined susceptibility changes, whereas χ-separation aims for increased biological specificity at the cost of reduced robustness in technically challenging regions. The improved performance of χ-separation_DL_ in the same region suggests that AI-assisted preprocessing may partially alleviate these limitations by stabilizing the source separation under clinically realistic acquisition conditions.

In the premotor cortex, R2* mapping revealed group differences that were broader in spatial extent than those detected by χ-separation, including effects in both PMd and PMv. R2* reflects a composite signal influenced by multiple microstructural factors, such as iron, myelin, and water content. While these factors are summed up in the same direction, making R2* highly sensitive to disease-related changes, the R2* value itself is limited by its biological specificity. In contrast, χ-separation explicitly targets the paramagnetic susceptibility component, providing a more iron-specific measure, at the cost of reduced sensitivity to subtle iron alterations, and vulnerability to artifacts. The absence of χ-separation effects in PMv therefore may not contradict the R2* findings but rather highlights the complementary nature of relaxation-based and source-separated susceptibility metrics in capturing different aspects of Parkinson-related cortical pathology.

The clinical relevance of χ-separation metrics was further supported by exploratory correlation analyses. In the PMd, χ_para_ correlated with disease duration, pointing towards its potential as a cortical progression marker. In subcortical regions, χ_QSM_ in the putamen and red nucleus showed complementary associations with MDS-UPDRS-III scores and disease duration. Although myelin concentrations are lower than iron in most of the deep gray matter nuclei, prior work suggested that myelin can contribute measurably to χ_QSM_^13^. Because χ_QSM_ aggregates effects from both iron accumulation and demyelination, χ_QSM_ may exhibit higher sensitivity to clinical status at the cost of reduced specificity to iron. Together with the χ_QSM_ observations, χ-separation_DL_ provides iron-specific complementary insights into disease-related susceptibility changes in PD.

Recent work illustrates the potential of susceptibility source separation in PD and related synucleinopathies. Dong et al. showed higher diagnostic power of χ_para_ vs. χ_QSM_ for differentiating PwPD with and without rapid-eye-movement (REM) sleep behavior disorder, using the APART-QSM approach^26^. Kiersnowski et al. used the DECOMPOSE framework in prodromal and overt α-synucleinopathies and observed that χ_para_ in the SN was already elevated in prodromal cases with abnormal DaT-SPECT^22^. Their study highlights early dopaminergic pathway vulnerability by cross-validating MRI with molecular imaging^22^. Shibata et al. applied QSM-ARCS to a large PD cohort and reported that χ_para_ correlated with both motor and cognitive performance^23^. They also developed machine learning models that distinguished PD with mild cognitive impairment from PD with normal cognition, underlining the potential of susceptibility metrics for patient stratification^23^. Yan et al. recently applied susceptibility source separation to the differential diagnosis of Parkinsonian syndromes in a cohort of PwPD and patients with multiple system atrophy^28^. They demonstrated that multiple system atrophy was characterized by marked paramagnetic increases in the Pu, GPe, GPi, and Thal, whereas PD exhibited more selective changes in the SN^28^. Unlike these studies, which employed model-based source separation algorithms, our framework integrates deep learning-based preprocessing with improved robustness to artifacts (Fig. 3) that may enhance biological interpretability.

It is noteworthy that R_2_* metrics did not reveal significant group differences for subcortical regions or clinically relevant correlations in our cohort. This finding emphasizes that while relaxation-based approaches remain widely used, they appear less sensitive to disease changes in this cohort and less biologically specific compared to source-separated susceptibility mapping.

Several considerations should be noted. None of the reported effects survived FDR-based correction for multiple comparisons. While several findings were statistically significant at the uncorrected level and showed biological plausibility, they should be considered preliminary. This further highlights the exploratory character of the study and the need for replication in larger and longitudinal datasets. The cross-sectional design precludes conclusions about longitudinal dynamics, and future studies will need to assess whether χ_para_ alterations track disease progression, predict phenotypic subtypes, or anticipate therapeutic response. Finally, susceptibility mapping in small nuclei such as the substantia nigra is vulnerable to noise, partial volume effects, and registration errors, which must be addressed in ongoing technical development. Despite these limitations, our study demonstrates the potential of χ-separation as an AI-assisted imaging biomarker platform for PD. By providing biologically specific markers of iron, χ-separation could be leveraged in multimodal frameworks that integrate imaging with genetic, molecular, and fluid biomarkers to refine disease stratification. Moreover, its sensitivity to cortical and subcortical changes makes it a candidate endpoint for clinical trials, particularly in prodromal or atypical Parkinsonian syndromes where diagnostic specificity remains a major challenge.

The development of AI-enabled susceptibility source separation carries several implications for PD research and care. First, the ability to isolate iron-related signals could enhance early detection by identifying subtle pathological changes in prodromal or at-risk individuals before overt clinical symptoms emerge. Second, source-specific susceptibility measures may serve as progression markers that can improve trial design, for example by enriching patient cohorts with rapid progressors or stratifying by underlying pathology. Third, χ-separation could provide objective, imaging-based endpoints for monitoring therapeutic interventions, including iron-targeting therapies and neuromodulatory approaches such as deep brain stimulation^29,30^. Finally, integrating χ-separation into multimodal pipelines that combine imaging with molecular and genetic biomarkers may accelerate the development of precision medicine approaches for PD. Together, these translational perspectives underscore that χ-separation is not only a methodological advance but also a potential enabler of more biologically grounded, clinically meaningful imaging biomarkers in PD.

## Methods

### Recruitment and clinical characteristics

The present study has been performed after receiving prior approval from the institutional review board of Johns Hopkins University, Baltimore, MD, USA (#00371335) and follows the revised version of the Declaration of Helsinki. All participants gave their informed written consent before any study-related activities were performed. We enrolled PwPD when the highest level of clinical diagnostic certainty, defined by the term *clinically established Parkinson’s disease* based on the revised diagnostic criteria of the Movement Disorders Society, was met^31^. We assessed demographic data, medical history, the current medication state (to calculate the levodopa-equivalent daily dosage, LEDD), and potential exclusion criteria (e.g., the presence of any other neurological comorbidity)^32,33^. Study participants were recruited via our outpatient clinics and from pre-existing cohorts. Trained movement disorders specialists performed standardized clinical assessments (e.g., MoCA) and a neurological examination in accordance with the MDS-UPDRS-III^34,35^.

### MRI acquisition

The participants underwent multi-contrast neuroimaging sessions on a 3 T MRI scanner equipped with a high-performance gradient system (maximum gradient strength: 95 mT/m; slew rate: 220 T/m/s; Ingenia Elition RX, Philips, Best, The Netherlands). Anatomical imaging protocols included T1-weighted (T1w) multi-echo MPRAGE^36^ (resolution = 1×1×1 mm^3^, TR = 14 ms, TE = 2.1 ms, echo spacing = 3.1 ms, number of echoes = 4, TI = 900 ms, FoV = 240 × 240 ×180 mm^3^, flip angle = 9°, total acquisition time = 2 min 54 sec) and T2-weighted FLAIR^37^ (resolution = 1×1×1 mm^3^, TR = 4800 ms, TE = 328 ms, TI = 1650 ms, FoV = 250×250×176 mm^3^, total acquisition time = 4 min 24 sec). For R_2_* ( = 1/T2*) and phase mapping, multi-echo gradient echo (MEGE) imaging was performed with the following parameters, with similar parameters with recently recommended protocols^38^: resolution = 0.8 × 0.8 × 0.8 mm^3^, TR = 40 ms, first TE = 7.0 ms, echo spacing = 7.0 ms, number of echoes = 5, FoV = 220 × 220 × 176 mm^3^, total acquisition time = 6 min. For R_2_ ( = 1/T2) mapping, 2D multi-echo turbo-spin echo (METSE) sequence was applied with the following protocol: in-plane resolution = 1 × 1 mm^2^, slice thickness = 2 mm, TR = 7000 ms, first TE = 20 ms, echo spacing = 20 ms, number of echoes = 4, FoV = 240 × 180 × 150 mm^3^, total acquisition time = 5 min 22 sec.

### MRI postprocessing and analyses

**Fig. 4:**
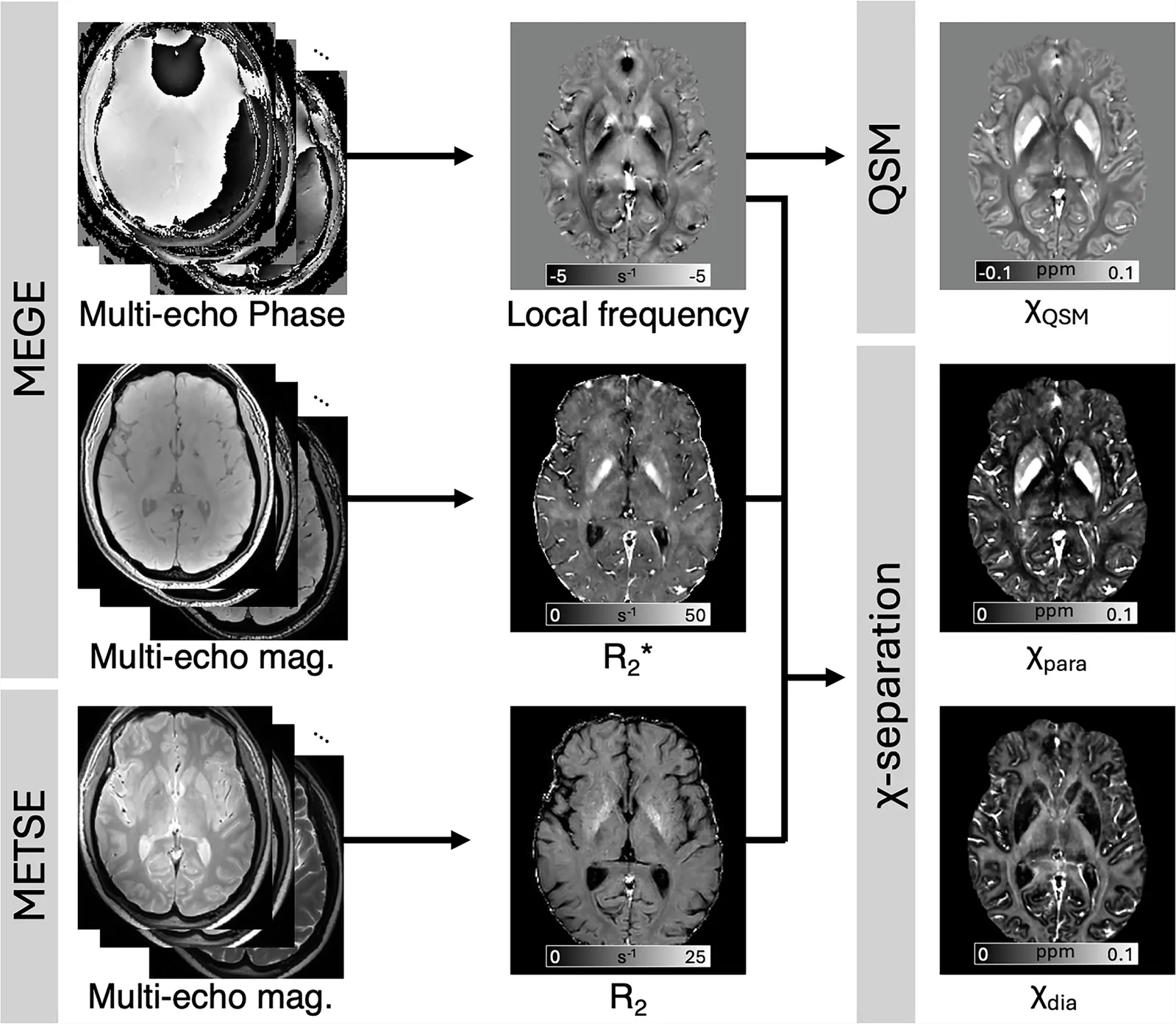
Schematic of the data processing pipeline. Local frequency, R_2_*, and R_2_ maps were derived from MEGE phase and magnitude data, and from METSE magnitude data, respectively. QSM map was reconstructed from the local frequency map, while χ-separation maps (χ_para_ and χ_dia_) were estimated using both the frequency map and the R_2_′ map (R_2_′ = R_2_* − R_2_). MEGE: multi-echo gradient-echo. METSE: multi-echo turbo spin-echo. QSM: Quantitative susceptibility mapping. χ_QSM:_ total magnetic susceptibility derived from QSM. χ_para/dia_: paramagnetic/diamagnetic component derived from χ-separation imaging.

**Fig. 5:**
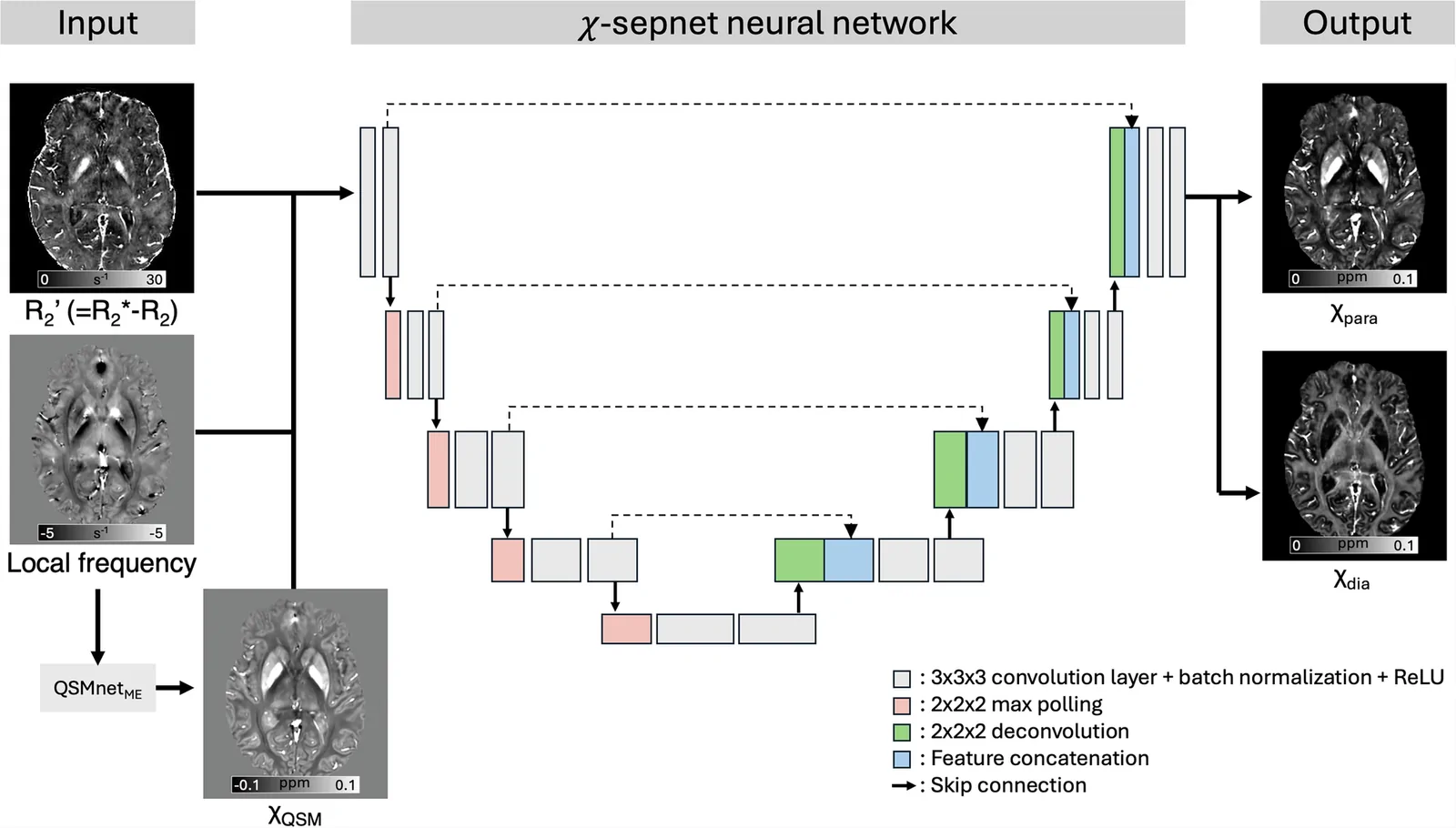
The 3D U-net architecture of χ-sepnet^24^. The network was designed with 18 convolutional layers (kernel size = 3×3×3), each combined with batch normalization and ReLU layers, 4 max pooling layers (kernel size = 2×2×2), 4 deconvolutional layers (kernel size = 2×2×2), 4 feature concatenations, and 4 skip connections for spatial information preservation. Inputs are 3-channel 3D maps (concatenation of R_2_’, local frequency, and χ_QSM_ [reconstructed from local frequency using QSMnet_ME_] maps), and the outputs are 2-channel (χ_para_ and χ_dia_). *ReLU: rectified linear unit*.

For R_2_* mapping, magnitude signals of MEGE data were denoised along the echo dimension using Marchenko-Pastur principal component analysis^39^ (with 5 × 5 × 5 kernel size), and a single exponential model was voxel-wisely fitted to the denoised magnitude signal decay. Multi-echo phase images from MEGE sequence underwent sequential multi-step processing^38^: spatial unwrapping using ROMEO^40^, SNR-based echo combining^41^, and background field removal using V-SHARP^42^, producing local frequency maps. QSM maps (χ_QSM_) were reconstructed from local frequency maps using a conventional QSM algorithm, named MEDI+0^43^. χ_QSM_ values were defined relative to water, by setting the mean susceptibility of cerebrospinal fluid (CSF) to zero. For R_2_ estimation, multi-echo spin echo signal decays were computer-simulated, considering stimulated echo effects^44^, and subsampled according to the actual TEs of METSE acquisition. Actual RF pulses were used to simulate the slice profile for the computation. Acquired METSE signals were matched to simulated signals via dictionary matching for R_2_ mapping. A rigid-body transformation matrix from METSE to MEGE space was computed using the first echo images of both contrasts and applied to align the R_2_ map to the R_2_* map^45^. Subsequently, R_2_’ was calculated by subtracting R_2_ from R_2_* voxel-wise. Negative R_2_’ values were truncated to zero, to enforce physically plausible constraints (i.e., R_2_* ≥ R_2_). To achieve enhanced boundaries of subcortical structures, the QSM map was rigidly registered to T1w MPRAGE image to create a T1w-QSM hybrid contrast^46,47^ following Min et al.^25^. The registration utilized the transformation matrix calculated from aligning the first echo magnitude image of MEGE to the T1w image. From the inputs of R_2_’ and local frequency images, χ_para_ and χ_dia_ maps were reconstructed using both conventional optimization-based χ-separation_MEDI_ and DL-based χ-separation_DL_ algorithms. For χ-separation_DL_, a pre-trained neural network (χ-sepnet^24^) was employed. Overall processing pipelines for quantitative MRI parameters were illustrated in Fig. 4. χ-sepnet employed a 3D U-net neural network architecture, as utilized in QSMnet^24,48^ (see Fig. 5 for further details on the architecture). All the χ-separation reconstructions were performed using the publicly available chi-separation toolbox (https://github.com/SNU-LIST/chi-separation). For a more straightforward interpretation of paramagnetic and diamagnetic susceptibility signal as iron and myelin content, respectively, we defined χ_para_ and χ_dia_ as the absolute susceptibility of paramagnetic and diamagnetic sources, relative to water (i.e., χ_para_ > 0; χ_dia_ > 0; total susceptibility = χ_para_ − χ_dia_).

Sensorimotor function-related regions of interest (ROIs) were segmented using the HMAT atlas (for cortical gray matter, cGM), which includes six cortical regions: M1, S1, SMA, preSMA, PMd, and PMv^49^. The T1w MPRAGE image was non-linearly warped to the template space, and the corresponding segmentations (in the template) were transformed back to the original MPRAGE space using inverse transformations. HMAT-derived cGM segmentations were refined by intersecting with cGM and white matter (WM) masks generated from MPRAGE images using SynthSeg^50,51^. The refined segmentations were registered to MEGE space using pre-calculated linear transformations between MPRAGE and MEGE spaces. For deep gray matter regions (dGM) segmentation, the T1w-QSM hybrid images were warped to the hybrid MuSus-100 atlas^47^. The thalamic nuclei were grouped into pulvinar and non-pulvinar regions (left and right, separately), resulting in a total of 12 dGM ROIs: Cau, Pu, GPe, NAcc, SNc, SNr, RN, VP, GPi, STN, Pulv, Thal. To mitigate paramagnetic effects from deoxyhemoglobin, veins were segmented, as proposed by Kim et al.^52^, and excluded from the ROI analyses. Finally, the mean and standard deviation of QSM, χ_para_, and R_2_* values were calculated for each ROI. From these, we calculated the hemisphere-averaged mean values and selected the ROIs contralateral to the clinically more affected side for subsequent analyses.

### Statistics

Descriptive statistics are reported as mean ± standard deviations (SD) unless stated otherwise. Outlier detection and correction were performed using an interquartile range (IQR)-based approach, excluding data points lying beyond 1.5×IQR from the first or third quartile. This procedure led to varying degrees of freedom in the subsequent analysis of covariance (ANCOVA) analyses, depending on whether and how many datasets were excluded due to outlier correction. Statistical analyses were conducted using custom in-house R scripts including data visualization. For our primary hypothesis, we performed ANCOVAs to compare neuroimaging measures between PwPD and HCs, with ROI-specific values of QSM, χ_para_, and R_2_* as dependent variables. Age and sex were used as covariates in all group comparisons to control for potential confounding effects. To identify associations among our neuroimaging measures and between our neuroimaging measures and clinical data (e.g., disease duration, MDS-UPDRS-III scores, LEDD), we performed Pearson’s correlation analyses. Correlation coefficients were computed for metrics and regions showing statistically significant or trend-level group differences. In this report, we considered the statistical analyses, given the multiparametric nature of approach, to be of a fully exploratory nature. For group comparisons, relevant significant P-values were corrected for multiple comparisons using the False Discovery Rate (FDR) according to the Benjamini-Hochberg procedure, and the corrected values are reported alongside the uncorrected ones. FDR correction was applied separately for each susceptibility metric (χ_QSM_, χ_para,DL_, χ_para,MEDI_, and R2*) and within anatomically defined ROI groups (cortical motor ROIs and subcortical ROIs). All correlative analyses are presented uncorrected. All above-stated analyses were performed only when necessary assumptions (e.g., a normal distribution, homogeneity of variance) were met.

## Supplementary information

ChiSep_SupplementaryMaterial_rev1

## Data Availability

The datasets generated and/or analyzed during the current study are not publicly available due to data protection regulations and institutional ethics restrictions, as the data contain potentially identifiable clinical and imaging information. However, the datasets are available from the corresponding author on reasonable request and subject to approval by the responsible ethics committee and data transfer agreements in accordance with applicable data protection laws (including GDPR).

## Code availability

The code used for the analyses in this study is available from the corresponding author upon reasonable request.
