# Supplementary material for: In-vivo iron mapping in patients with Parkinson’s disease using deep learning-based susceptibility source separation MRI
Source: NPJ Parkinsons Dis. 2026 May 27;12:200. doi: 10.1038/s41531-026-01384-x (PMC13493794; doi:10.1038/s41531-026-01384-x)
Supplement: Supplementary file 1 [file 41531_2026_1384_MOESM1_ESM.docx]

**Supplementary Table 1. Regional group comparisons of hemisphere-averaged and unilateral (on the clinically more affected side) diamagnetic quantitative susceptibilities^†^ (in ppm).**

|  |  | **PwPD** | | | **HCs** | | | **F(df)^a^** | **P-value^a^**  **∣**  **P-value_FDR_** |
| --- | --- | --- | --- | --- | --- | --- | --- | --- | --- |
| **hemisphere-averaged** | | | | | | | | | |
| **χ_dia,DL_** | M1 | 0.019 | ± | 0.003 | 0.017 | ± | 0.002 | F(1, 46) = 3.12 | .084 ∣ .883 |
|  | S1 | 0.020 | ± | 0.003 | 0.019 | ± | 0.002 | F(1, 46) = 3.79 | .058 ∣. 878 |
|  | SMA | 0.017 | ± | 0.001 | 0.017 | ± | 0.003 | F(1, 41) = 1.07 | .306 ∣ .765 |
|  | preSMA | 0.017 | ± | 0.002 | 0.016 | ± | 0.002 | F(1, 45) = 1.41 | .241 ∣ .765 |
|  | PMd | 0.018 | ± | 0.002 | 0.018 | ± | 0.002 | F(1, 44) = 0.15 | .702 ∣ .576 |
|  | PMv | 0.018 | ± | 0.001 | 0.018 | ± | 0.002 | F(1, 43) = 0.07 | .799 ∣ .878 |
| **χ_dia,MEDI_** | M1 | 0.027 | ± | 0.003 | 0.027 | ± | 0.002 | F(1, 40) = 0.53 | .472 ∣ .878 |
|  | S1 | 0.029 | ± | 0.005 | 0.028 | ± | 0.003 | F(1, 41) = 1.12 | .295 ∣ .878 |
|  | SMA | 0.029 | ± | 0.005 | 0.029 | ± | 0.005 | F(1, 43) = 0.04 | .852 ∣ .913 |
|  | preSMA | 0.028 | ± | 0.005 | 0.025 | ± | 0.007 | F(1, 47) = 1.67 | .203 ∣ .968 |
|  | PMd | 0.030 | ± | 0.004 | 0.028 | ± | 0.002 | F(1, 45) = 2.24 | .141 ∣ .532 |
|  | PMv | 0.029 | ± | 0.002 | 0.029 | ± | 0.002 | F(1, 42) = 0.21 | .650 ∣ .552 |
| **clinically more affected side** | | | | | | | | | |
| **χ_dia,DL_** | M1 | 0.019 | ± | 0.003 | 0.017 | ± | 0.002 | F(1, 44) = 2.56 | .117 ∣ .883 |
|  | S1 | 0.019 | ± | 0.002 | 0.019 | ± | 0.002 | F(1, 43) = 1.81 | .186 ∣. 878 |
|  | SMA | 0.017 | ± | 0.003 | 0.017 | ± | 0.003 | F(1, 45) = 0.90 | .349 ∣ .765 |
|  | preSMA | 0.017 | ± | 0.002 | 0.016 | ± | 0.002 | F(1, 44) = 1.10 | .301 ∣ .765 |
|  | PMd | 0.020 | ± | 0.004 | 0.018 | ± | 0.002 | F(1, 46) = 2.56 | .117 ∣ .576 |
|  | PMv | 0.018 | ± | 0.002 | 0.018 | ± | 0.002 | F(1, 43) = 0.02 | .891 ∣ .878 |
| **χ_dia,MEDI_** | M1 | 0.026 | ± | 0.003 | 0.027 | ± | 0.002 | F(1, 39) = 1.28 | .265 ∣ .878 |
|  | S1 | 0.029 | ± | 0.006 | 0.028 | ± | 0.003 | F(1, 40) = 0.90 | .348 ∣ .878 |
|  | SMA | 0.028 | ± | 0.007 | 0.029 | ± | 0.005 | F(1, 44) = 0.33 | .567 ∣ .913 |
|  | preSMA | 0.028 | ± | 0.005 | 0.025 | ± | 0.007 | F(1, 46) = 2.68 | .109 ∣ .968 |
|  | PMd | 0.030 | ± | 0.006 | 0.028 | ± | 0.002 | F(1, 44) = 1.86 | .180 ∣ .532 |
|  | PMv | 0.028 | ± | 0.003 | 0.029 | ± | 0.002 | F(1, 41) = 0.07 | .794 ∣ .552 |

^†^ Mean ± standard deviation of susceptibility parameters in predefined motor cortical regions of interest for patients with Parkinson’s disease (PwPD) and healthy controls (HCs). **^a^** Reported statistics are F-values with corresponding degrees of freedom and p-values from univariate ANCOVAs (accounting for sex and age as covariates). *DL: deep learning-based reconstruction. MEDI: morphology-enabled dipole inversion. χ_dia,DL_: diamagnetic component derived from deep-learning χ-separation. Χ_dia,MEDI_: diamagnetic component derived from MEDI-based χ-separation. HCs: healthy controls. PwPD: patients with Parkinson’s disease. M1: primary motor cortex. S1: primary somatosensory cortex. SMA: supplementary motor area. preSMA: pre-supplementary motor area. PMd: dorsal premotor cortex. PMv: ventral premotor cortex.*

**Supplementary Table 2. Regional group comparisons of hemisphere-averaged and unilateral (on the clinically more affected side) subcortical diamagnetic quantitative susceptibility metrics^†^ (in ppm).**

|  |  | **PwPD** | | | **HCs** | | | **F(df) ^a^** | **P-value ^a^**  **∣**  **P-value_FDR_** |
| --- | --- | --- | --- | --- | --- | --- | --- | --- | --- |
| **hemisphere averaged** | | | | | | | | | |
| **χ_dia,DL_** | Cau | 0.014 | ± | 0.001 | 0.014 | ± | 0.003 | F(1, 44) = 0.54 | .467 ∣ .980 |
|  | Pu | 0.015 | ± | 0.002 | 0.014 | ± | 0.002 | F(1, 44) = 6.98 | .011 ∣ .720 |
|  | GPe | 0.011 | ± | 0.002 | 0.012 | ± | 0.003 | F(1, 44) = 2.53 | .119 ∣ .720 |
|  | NAcc | 0.029 | ± | 0.003 | 0.030 | ± | 0.005 | F(1, 47) = 1.30 | .261 ∣ .980 |
|  | SNc | 0.008 | ± | 0.002 | 0.009 | ± | 0.004 | F(1, 45) = 0.66 | .419 ∣ .288 |
|  | SNr | 0.011 | ± | 0.005 | 0.011 | ± | 0.005 | F(1, 46) = 0.15 | .702 ∣ .812 |
|  | RN | 0.019 | ± | 0.004 | 0.020 | ± | 0.004 | F(1, 44) = 0.12 | .733 ∣ .860 |
|  | VP | 0.022 | ± | 0.009 | 0.020 | ± | 0.009 | F(1, 45) = 0.58 | .451 ∣ .980 |
|  | GPi | 0.015 | ± | 0.003 | 0.017 | ± | 0.005 | F(1, 45) = 2.80 | .101 ∣ .720 |
|  | STN | 0.019 | ± | 0.005 | 0.018 | ± | 0.003 | F(1, 45) = 0.95 | .335 ∣ .720 |
|  | Thal | 0.032 | ± | 0.003 | 0.033 | ± | 0.003 | F(1, 47) = 0.43 | .517 ∣ .812 |
|  | Pulv | 0.018 | ± | 0.003 | 0.018 | ± | 0.003 | F(1, 46) = 0.87 | .357 ∣ .720 |
| **χ_dia,MEDI_** | Cau | 0.024 | ± | 0.005 | 0.022 | ± | 0.005 | F(1, 45) = 2.76 | .104 ∣ 1.000 |
|  | Pu | 0.032 | ± | 0.007 | 0.028 | ± | 0.008 | F(1, 45) = 2.68 | .108 ∣ 1.000 |
|  | GPe | 0.027 | ± | 0.006 | 0.031 | ± | 0.014 | F(1, 45) = 2.58 | .115 ∣ 1.000 |
|  | NAcc | 0.044 | ± | 0.016 | 0.044 | ± | 0.018 | F(1, 47) = 0.00 | .980 ∣ 1.000 |
|  | SNc | 0.022 | ± | 0.011 | 0.028 | ± | 0.025 | F(1, 46) = 0.87 | .356 ∣ 1.000 |
|  | SNr | 0.025 | ± | 0.010 | 0.033 | ± | 0.023 | F(1, 44) = 1.77 | .190 ∣ 1.000 |
|  | RN | 0.041 | ± | 0.011 | 0.048 | ± | 0.028 | F(1, 44) = 0.60 | .444 ∣ 1.000 |
|  | VP | 0.050 | ± | 0.021 | 0.045 | ± | 0.020 | F(1, 45) = 0.45 | .506 ∣ 1.000 |
|  | GPi | 0.035 | ± | 0.009 | 0.040 | ± | 0.017 | F(1, 44) = 1.12 | .296 ∣ 1.000 |
|  | STN | 0.035 | ± | 0.015 | 0.033 | ± | 0.014 | F(1, 46) = 0.15 | .696 ∣ 1.000 |
|  | Thal | 0.040 | ± | 0.006 | 0.040 | ± | 0.010 | F(1, 45) = 0.02 | .901 ∣ 1.000 |
|  | Pulv | 0.020 | ± | 0.006 | 0.021 | ± | 0.006 | F(1, 45) = 0.44 | .511 ∣ 1.000 |
| **clinically more affected side** | | | | | | | | | |
| **χ_dia,DL_** | Cau | 0.015 | ± | 0.002 | 0.014 | ± | 0.003 | F(1, 45) = 2.97 | .092 ∣ .980 |
|  | Pu | 0.015 | ± | 0.002 | 0.014 | ± | 0.002 | F(1, 43) = 5.15 | .028 ∣ .720 |
|  | GPe | 0.011 | ± | 0.002 | 0.012 | ± | 0.003 | F(1, 43) = 3.23 | .080 ∣ .720 |
|  | NAcc | 0.029 | ± | 0.003 | 0.030 | ± | 0.005 | F(1, 44) = 1.16 | .288 ∣ .980 |
|  | SNc | 0.009 | ± | 0.003 | 0.009 | ± | 0.004 | F(1, 45) = 0.02 | .887 ∣ .288 |
|  | SNr | 0.011 | ± | 0.005 | 0.011 | ± | 0.005 | F(1, 44) = 0.21 | .647 ∣ .812 |
|  | RN | 0.019 | ± | 0.005 | 0.020 | ± | 0.004 | F(1, 43) = 0.05 | .823 ∣ .860 |
|  | VP | 0.022 | ± | 0.011 | 0.020 | ± | 0.009 | F(1, 44) = 0.49 | .487 ∣ .980 |
|  | GPi | 0.016 | ± | 0.003 | 0.017 | ± | 0.005 | F(1, 43) = 1.20 | .280 ∣ .720 |
|  | STN | 0.020 | ± | 0.007 | 0.018 | ± | 0.003 | F(1, 45) = 1.95 | .169 ∣ .720 |
|  | Thal | 0.032 | ± | 0.003 | 0.033 | ± | 0.003 | F(1, 46) = 0.96 | .333 ∣ .812 |
|  | Pulv | 0.018 | ± | 0.004 | 0.018 | ± | 0.003 | F(1, 46) = 0.68 | .413 ∣ .720 |
| **χ_dia,MEDI_** | Cau | 0.024 | ± | 0.006 | 0.022 | ± | 0.005 | F(1, 44) = 2.68 | .109 ∣ 1.000 |
|  | Pu | 0.031 | ± | 0.008 | 0.028 | ± | 0.008 | F(1, 44) = 2.20 | .146 ∣ 1.000 |
|  | GPe | 0.026 | ± | 0.006 | 0.031 | ± | 0.014 | F(1, 44) = 2.86 | .098 ∣ 1.000 |
|  | NAcc | 0.043 | ± | 0.016 | 0.044 | ± | 0.018 | F(1, 46) = 0.01 | .939 ∣ 1.000 |
|  | SNc | 0.024 | ± | 0.013 | 0.028 | ± | 0.025 | F(1, 46) = 0.19 | .664 ∣ 1.000 |
|  | SNr | 0.028 | ± | 0.014 | 0.033 | ± | 0.023 | F(1, 45) = 0.26 | .614 ∣ 1.000 |
|  | RN | 0.041 | ± | 0.012 | 0.048 | ± | 0.028 | F(1, 44) = 0.47 | .498 ∣ 1.000 |
|  | VP | 0.048 | ± | 0.024 | 0.045 | ± | 0.020 | F(1, 44) = 0.21 | .649 ∣ 1.000 |
|  | GPi | 0.035 | ± | 0.010 | 0.040 | ± | 0.017 | F(1, 43) = 0.93 | .339 ∣ 1.000 |
|  | STN | 0.037 | ± | 0.017 | 0.033 | ± | 0.014 | F(1, 45) = 0.63 | .431 ∣ 1.000 |
|  | Thal | 0.040 | ± | 0.008 | 0.040 | ± | 0.010 | F(1, 44) = 0.02 | .893 ∣ 1.000 |
|  | Pulv | 0.019 | ± | 0.006 | 0.021 | ± | 0.006 | F(1, 43) = 3.17 | .082 ∣ 1.000 |

**^†^** All metrics are reported as mean ± standard deviation. ^a^ p-values were obtained using univariate ANCOVA (accounting for sex and age as covariates) per region and metric. Significant (uncorrected) p-values are highlighted in gray. *DL: deep learning-based reconstruction. MEDI: morphology-enabled dipole inversion. Χ_dia,DL_: paramagnetic component derived from deep-learning χ-separation. Χ_dia,MEDI_: paramagnetic component derived from MEDI-based χ-separation. HCs: healthy controls. PwPD: patients with Parkinson’s disease. Cau: caudate nucleus. Pu: putamen. GPe: external globus pallidus. GPi: internal globus pallidus. NAcc: nucleus accumbens. SNc: substantia nigra pars compacta. SNr: substantia nigra pars reticulata. RN: red nucleus. VP: ventral pallidum. STN: subthalamic nucleus. Thal: thalamus. Pulv: pulvinar.*
